# Wild cognition – linking form and function of cognitive abilities within a natural context

**DOI:** 10.1016/j.cobeha.2022.101115

**Published:** 2022-03-02

**Authors:** Birgit Szabo, Anyelet Valencia-Aguilar, Isabel Damas-Moreira, Eva Ringler

**Affiliations:** 1 Division of Behavioural Ecology, Institute of Ecology and Evolution, University of Bern, Bern, Switzerland; 2 Behavioural Ecology, Faculty of Biology, Bielefeld University, Bielefeld, Germany

## Abstract

Interest in studying cognitive ecology has moved the field of animal cognition into the wild. Animals face many challenges such as finding food and other resources, avoiding and deterring predators and choosing the best mate to increase their reproductive success. To solve these dilemmas, animals need to rely on a range of cognitive abilities. Studying cognition in natural settings is a powerful approach revealing the link between adaptive form and biological function. Recent technological and analytical advances opened up completely new opportunities and research directions for studying animal cognition. Such innovative studies were able to disclose the variety in cognitive processes that animals use to survive and reproduce. Cognition indeed plays a major role in the daily lives of wild animals, in which the integration of many different types of information using a diverse range of cognitive processes enhances fitness.

## Introduction

The research field of animal cognition is going through a remarkable change from a largely lab focused approach towards an increasing appreciation of field studies in wild animals. In the wild, animals need to constantly adapt their behaviour to the changing natural and social environment, which might have given rise to distinct cognitive abilities across animal taxa. Finding food and other crucial resources is one of the main challenges for most animals. While some species have to migrate vast distances to reach these resources [1], non-migratory species have to be able to cope with harsh winters or hot summers in which resources might be scarce [2]. Another challenge is the need to be aware of predators, be vigilant and detect dangers early to escape [3]. Along with finding resources and surviving, individuals also need to pass their genes on to the next generation. The ability to find the best possible mate and successfully raise offspring is also underlined by cognitive skills such as a longer memory for resource locations [4]. Each set of challenges thus requires different cognitive abilities, and individuals need to learn, process and retain information quickly and efficiently to overcome problems. The dilemma of find resources, avoid predation and increase reproduction are common across all taxa, but the cognitive skills used to solve them can vary.

Controlled lab studies are able to disentangle the factors affecting different cognitive processes in animals, but fail to provide a strong link between a cognitive ability and its function in the wild, for which the behaviour has evolved [5]. Studies under natural conditions provide an understanding of the environmental factors that shaped cognition, revealing their function in an evolutionary framework. However, proper manipulation and control of experimental settings can be challenging or even impossible [6]. The change towards ‘wild cognition’ was possible mostly due to the recent technological advancements, such as tracking devices, high-quality video cameras, electronic portable devices, or novel molecular and data analysis methods. These tools allow studying animal cognition directly in the wild [6] and reveal the impact of cognition on individual fitness through changes in survival or reproductive success. Moreover, studies on wild animals have additional advantages: (1) they are more likely to capture the full range of behaviour in response to the natural environment and (2) they make it possible to study a larger sample size because the number of animals is not restricted by laboratorial capacity nor other logistical issues related to limited space.

**Figure 1:**
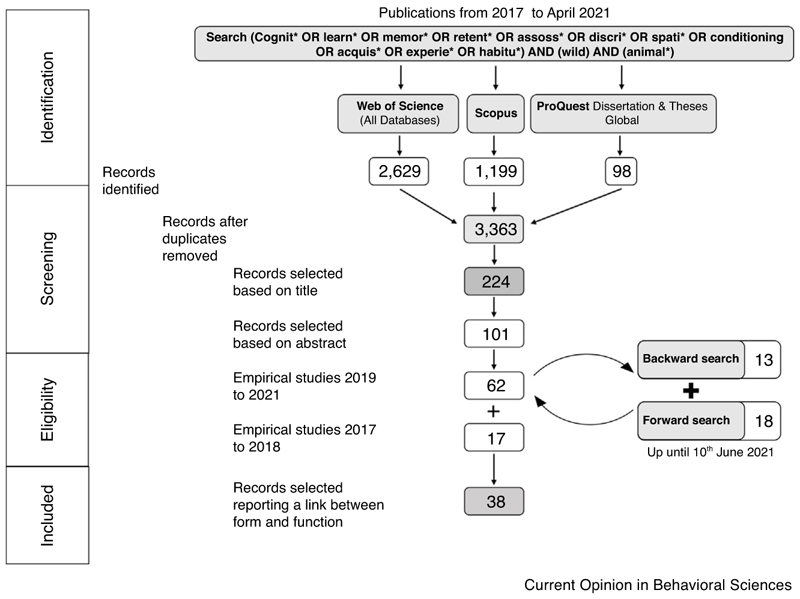
PRISMA diagram describing the systematic search performed for this review. We used the same search term to search three data bases (Web of Science, Scopus and ProQest Dissertation and Thesis Global) to identify published literature regarding cognition in wild animals. 3363 records were screened and 224 selected based on their title. Further screening of the abstracts reduced the number of selected records to 101. As this review focuses on the most recent advances in the field we only deemed studies from the last five years (2017–2021) as eligible and only included studies that clearly provided a link between the tested cognitive ability and its function in wild animals (*N* = 38). For a more detailed description see the provided electronic supplementary material.

**Figure 2:**
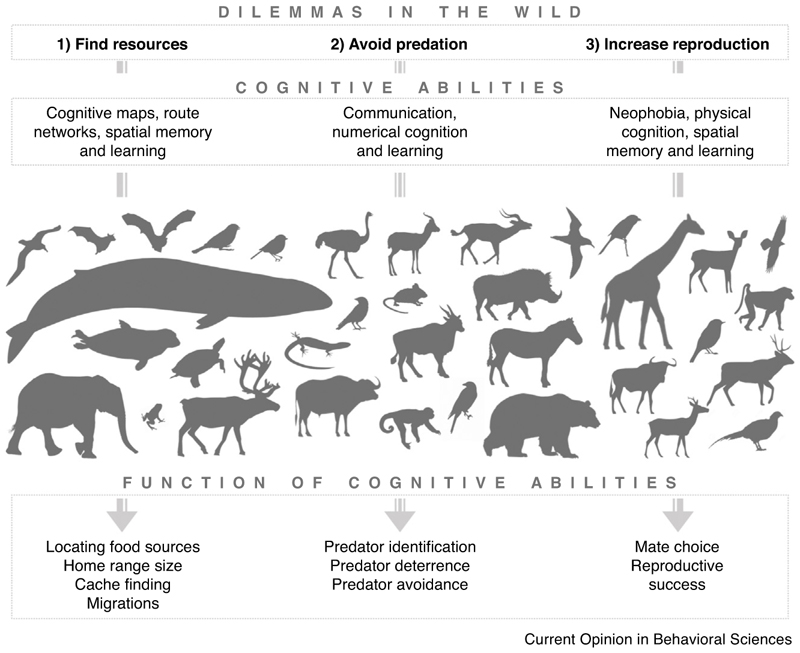
Graphical summary of the reviewed literature. We identified three major dilemmas animals face in the wild under which we summarised the selected literature in the main text. In the context of finding resources, animals use learning and memory to develop cognitive maps and route networks, to establish their home range, find their caches and accomplish migrations. In the context of avoiding predators, animals use learning, communication and numerical cognition to identify, deter and avoid predation. Lastly, in the context of reproduction, animals - rely on novelty recognition, associative learning, spatial memory and physical cognition to choose mates and increase their reproductive success. Overall, studies are mostly conducted in mammals and birds but we were also able to include studies in reptiles and an amphibian. Outline credits: Bat – M. Ingala; Capuchin – S. Werning; Wale – C. Huh; Raven – D. Bakken and T. M. Keesey; Wildebeest, Bovid, and Oryx – J. A. Venter, H. H. T. Prins, D. A. Balfour, R. Slotow and T. M. Keesey; Gazelle – R. Groom; Sparrow – A. Butko. Link to license: https://creativecommons.org/licenses/by/3.0/. Outlines were downloaded from http://www.phylopic.org/.

In this short review, we highlight and summarise some of the most recent (within the past five years) empirical studies that provide unique and novel insights into the form and function of cognitive abilities as they are used by animals in the wild. To provide the most unbiased overview, we performed a systematic literature search (electronic supplementary material; Figure 1). The selected studies provide insights into the diversity of cognitive abilities that evolved specifically to solve the challenges of finding resources, avoiding predators, and increasing reproductive success (Figure 2).

### Dilemma 1: navigating through space to find resources

Locating resources all year round is essential for survival [3]. Long-distance migrations in the pursuit of resources are among the most impressive examples, but good navigation is important across different spatial scales. How animals accomplish these movements has become much clearer through tracking technology such as GPS and long-term study data analysis from sometimes hundreds of individuals followed over months or years [6,7,8^••^]. Such data revealed that some mammals (African elephant, *Loxodonta africana*; Egyptian fruit bat, *Rousettus aegyptiacus*) navigate between resources using unique routes when they are familiar with the area [7,8^••^]. The use of such unique routes is characteristic for Euclidean cognitive maps, a map like mental representation of familiar space [5]. Another way to reach specific resources is the use of topological cognitive maps in which a system of routes connects resources [5]. Two recent studies followed chacma baboons (*Papio ursinus*) and bearded capuchin monkeys (*Sapajus libidinosus*) for several 100 days to record their location via GPS from which their daily movement was reconstructed. The resulting movement patterns revealed that these two primate species depend on such route systems when foraging [9,10]. Painted turtles (*Chrysemys picta*) also use routes to navigate across terrestrial habitat. Translocating individuals demonstrated that these routes are learnt during a critical period early in life, navigation is independent of season and each adult turtle uses a specific route [11^••^].

A basic mechanism for the establishment of maps or routes is a good memory of resource locations but the involvement of spatial memory in large scale movement has only recently been demonstrated. In these studies, GPS tracking, telemetry data or RFID/PIT systems were used to follow the movement of individuals (from a few to multiple hundreds) over weeks [12], months [13] or many years [1,14,15]. Birds (albatross, *Thalassarche melanophrys;* manx shearwater, *Puffinus puffinus*) [13,14] and mammals (deer, *Capreolus capreolus;* blue whales, *Balaenoptera musculu;* barren-ground caribou, *Rangifer tarandus granti*) [12,1,15] use spatial memory to cross large distances in the pursuit of food (fishing boats, winter feeding stations or krill patches) [12,1], to return home [14] or to reach their calving sites [15]. More and more research takes advantage of modelling techniques that are able to disentangle the different cognitive mechanisms applied while animals move through space [6]. The simulated movement of artificial individuals that rely on different mechanisms is compared to real life tracking data (GPS or telemetry) to reveal which mechanism is most likely underlying the movement of real animals. Such an approach demonstrated that zebra, *Equus burchelli antiquorum*, and mule deer, *Odocoileus hemionus*, rely on memory to accomplish their migrations [16,17]. In this way, researchers have also been able to show that home range size can be determined by learning and memory (grizzly bear, *Ursus arctos*) [18] and that new resources are discovered at a 20-fold greater rate through social learning compared to individual learning (e.g.: black-capped chickadees, *Poecile atricapillus*) [19]. Spatial memory is, however, equally important at smaller scales. Birds (black-capped chickadees), for example, heavily rely on memory to find stored food during winter and those individuals with better memory are more likely to survive through the first winter [2].

Naturally, such knowledge of the environment is acquired at an earlier point in life. Close tracking of juveniles, starting from when they first become independent, showed that they need to familiarise themselves with their nearby environment to develop cognitive maps (Egyptian fruit bats) [20] and to improve on their foraging routes (pheasants, *Phasianus colchicus;* grey seal, *Halichoerus grypus*) [21,22].

Current research has also shown that animals might rely on multiple sources of information for navigation and orientation in the environment. For example, female Bechstein’s bats (*Myotis bechsteinii*) rely on social and individual learning and use spatial memory to find suitable roost locations when provided with artificial roost boxes [23]. African elephants use cognitive maps when moving in familiar space but rely on a route networks in unfamiliar environments [7]. Therefore, it is likely that animals might not just rely on a single, but rather a combination of multiple cognitive processes (e.g. memory, social and individual learning, maps and routes), to effectively locate resources. The use of modern technology (e.g. data loggers and data processing tools) to reveal the diverse types of information animals use to navigate through space and time under natural settings will surely advance our understanding of the adaptive value of cognition.

### Dilemma 2: avoiding predation

Predator avoidance is among the many challenges animals face on a daily basis [3]. Animals might hide, flee, reduce activity or seek safety in numbers to escape predation [3]. Some species, however, try to actively deter a predator using mobbing behaviour. Larger mobs are safer and more effective in chasing the predator away and some birds but not all [24] are able to recognise the larger mob to join (Numerical cognition: jackdaw, *Corvus monedula;* great tit) [25,26]. Learning can be an important component in the development of mobbing as shown in fledgling blue tits (*Cyanistes caeruleus*) that mainly produce incomplete mobbing behaviour when inexperienced [27].

Many species also produce alarm calls to communicate and advertise the presence of a predator. Such calls can contain specific information as to the identity of the predator. These calls evoke a search image of the specific predator in the receiver which cannot be evoked by other alarm calls (Japanese tit, *Parus minor*) [28]. Some species even eavesdrop on the alarm calls of other, sympatric species with which they share predators. Birds might eavesdrop on other birds (coal tits, *Periparus ater*, from Japanese tit) [29^••^], lizards might eavesdrop on birds (Kalahari tree skinks, *Trachylepis spilogaster*, from sociable weavers, *Philetairus socius*) [30^•^] and whole herbivore communities selectively use each other’s’ alarm calls [31–33]. Such eavesdropping enables animals to become more vigilant and escape to safety. Although most studies listed here did not investigate how animals acquire the knowledge of which alarm calls are relevant to them, learning can be an important mechanism [30^•^].

Numerical cognition can also play a role in general alarm calling. Australian magpies (*Gymnorhina tibicen*) respond more strongly to heterospecific alarm calls from caller pairs, again demonstrating an ability to discriminate quantities, but the identity of the heterospecific species does not matter [33]. Contrary to magpies, common ravens (*Corvus corax*) do not take the number of callers into account but become more vigilant in response to calls from experienced adults rather than inexperienced juveniles, revealing the importance of some components of information (age or experience) that are transmitted through the calls [34].

The examples above demonstrate how different species communicate important information and eavesdrop on each other to avoid predators or deter them, however, appropriately processing visual information is also important. Red-backed shrikes (*Lanius collurio*), for example, attack a common kestrel (*Falco tinnunculus*) dummy less when the head is not at the top of the body a sign of selectivity when it comes to threat recognition [35].

Overall, studying individuals in the wild can be extremely powerful in the context of predator avoidance, as cognitive skills in detecting and identifying predators as well as communicating their presence can be directly linked to individual survival. In this context, manipulating the available information about potential predators under natural conditions provides clear evidence of what is communicated and what is important to elicit anti-predator responses even across species. How species acquire the ability to understand heterospecific signals is a valuable and interesting avenue for future research in this area.

### Dilemma 3: increasing reproductive success

Cognitive processes such as learning might be modulated by both natural [2] and sexual selection [36]. As such, selected cognitive processes can be crucial not just for survival but also for reproductive success. Individuals with better associative learning performance, for example, may achieve higher fitness, but most of the supporting evidence comes from lab experiments [37]. Recent data from studies on wild house sparrows (*Passer domesticus*), New Zealand robins (*Petroica longipes*) and great tits show that parents with lower neophobia (spending less time near a novel apparatus before successfully solving a problem) [38], better physical cognition (e.g. problem-solving success through string pulling [39]), and a better spatial memory [40], respectively, provide better for their offspring and raise more chicks. Interestingly, dominant male African striped mice (*Rhabdomys pumilio*) were faster than philopatric and solitary males in learning to open a door to escape a box possibly because of their need to return to their nest to guard females and offspring [41^•^]. Experience and learning can also play an essential role in identifying and distinguishing potential mating partners from rivalling individuals. A study using a Neotropical poison frog (*Allobates femoralis*) found that younger individuals were more likely to attack a non-threatening model during acoustic playback than older, more experienced, frogs [42].

Better cognitive skills (e.g. longer memory or less/more neophobia) might improve males’ ability to acquire and maintain more and/or better resources and hence, females choosing those males may obtain both direct (shelter, food) and indirect benefits (their offspring would inherit those skills) [36]. Indeed, females of the mountain chickadee base their mate choice on males’ spatial cognitive skill, since males with better spatial learning and memory will improve both female and offspring fitness [4]. Moreover, in great tits, extra-pair paternity is related to innovativeness and neophilia. Both traits may facilitate the investment into extra-pair mating attempts at least in females [43].

Despite a widespread interest on how individual cognitive differences might lead to individual variation in fitness and reproductive success in wild populations, we show that studies mainly focus on birds and mammals at least in the last five years. Studies combining cognitive tests of wild animals with genetic pedigree analysis can provide direct evidence of the relationship between cognitive skill and reproductive success. Using this approach will make it possible to directly link an individuals’ cognitive skill to the number of offspring that are produced or surviving either within a season or even across a lifetime. We believe that such studies will become more common as genetic analysis tools become more widely available because costs are steadily decreasing.

## Conclusions

The strong focus on the function of cognitive processes rather than a demonstration of their existence has proven extremely important to better understand the adaptive significance of cognitive abilities that we observe in animals. Despite tremendous advances in linking form and function of cognitive abilities in wild animals, we see two main gaps that offer promising directions for future research. First, there is a bias when it comes to animal taxa used for cognitive studies — with a strong focus on birds and mammals. Within the last five years only two studies focused on investigating the cognitive abilities in wild reptiles [11^••^,30^•^], and using wild amphibians as models was even rarer [42,44]. Although we were unable to include studies in fishes or invertebrates as they did not pass our selection process, bees and ants cognitive ability and its’ function in the wild (spatial orientation and resource gathering), as an example, are exceedingly well studied [45,46]. We hope that the growing trend of testing non-mammal and non-bird species cognition will hopefully continue leading to a better understand of the evolutionary origins of distinct cognitive abilities. This will be key for our understanding of how cognitive abilities evolved and are shared across animals. Second, studies directly linking cognitive traits with individual fitness are still relatively scarce not just in the wild. This lack of research might be attributed to the long timeframe and the lack in availability of precise pedigree information required for such studies.

In this short review, we highlighted recent empirical studies mostly focusing on the last five years that provide novel insights into the diversity of cognitive abilities that evolved in response to challenges animals are facing in their natural environment. Furthermore, we also focused on studies demonstrating the importance of good cognitive skill when it comes to survival and reproductive success. These insights were made possible by the use of novel technology and analysis methods as well as long-term study data analysis boosting the field of cognitive ecology. We hope that this trend will continue further advancing our understanding of the link between the form and function of cognitive abilities in animals.

## Supplementary Material

Supplementary material

Supplementary reference table

## References

[1] Abrahms B, Hazen EL, Aikens EO, Savoca MS, Goldbogen JA, Bograd SJ, Jacox MG, Irvine LM, Palacios DM, Mate BR (2019). Memory and resource tracking drive blue whale migrations. Proc Natl Acad Sci U S A.

[2] Sonnenberg BR, Branch CL, Pitera AM, Bridge E, Pravosudov VV (2019). Natural selection and spatial cognition in wild food-caching mountain chickadees. Curr Biol.

[3] Rubenstein DR, Alcock J (2018). Animal Behaviour.

[4] Branch CL, Pitera AM, Kozlovsky DY, Bridge ES, Pravosudov VV (2019). Smart is the new sexy: female mountain chickadees increase reproductive investment when mated to males with better spatial cognition. Ecol Lett.

[5] Shettleworth SJ (2010). Cognition, Evolution, and Behavior.

[6] Lewis MA, Fagan WF, Auger-Méthé M, Frair J, Fryxell JM, Gros C, Gurarie E, Healy SD, Merkle JA (2021). Learning and animal movement. Front Ecol Evol.

[7] Presotto A, Fayrer-Hosken R, Curry C, Madden M (2019). Spatial mapping shows that some African elephants use cognitive maps to navigate the core but not the periphery of their home ranges. Anim Cogn.

[8] Toledo S, Shohami D, Schiffner I, Lourie E, Orchan Y, Bartan Y, Nathan R (2020). Cognitive map-based navigation in wild bats revealed by a new high-throughput tracking system. Science.

[9] de Raad AL, Hill RA (2019). Topological spatial representation in wild chacma baboons (*Papio ursinus*). Anim Cogn.

[10] Presotto A, Verderane MP, Biondi L, Mendonça-Furtado O, Spagnoletti N, Madden M, Izar P (2018). Intersection as key locations for bearded capuchin monkeys (*Sapajus libidinosus*) traveling within a route network. Anim Cogn.

[11] Krochmal AR, Roth TC, Simmons NT (2021). My way is the highway: the role of plasticity in learning complex migration routes. Anim Behav.

[12] Ranc N, Moorcroft PR, Ossi F, Cagnacci F (2021). Experimental evidence of memory-based foraging decisions in a large wild mammal. Proc Natl Acad Sci U S A.

[13] Collet J, Weimerskirch H (2020). Albatrosses can memorize locations of predictable fishing boats but favour natural foraging: albatross memory and boat predictability. Proc R Soc B Biol Sci.

[14] Padget O, Stanley G, Willis JK, Fayet AL, Bond S, Maurice L, Shoji A, Dean B, Kirk H, Juarez-Martinez I (2019). Shearwaters know the direction and distance home but fail to encode intervening obstacles after free-ranging foraging trips. Proc Natl Acad Sci U S A.

[15] Cameron MD, Joly K, Breed GA, Mulder CPH, Kielland K (2020). Pronounced fidelity and selection for average conditions of calving area suggestive of spatial memory in a highly migratory ungulate. Front Ecol Evol.

[16] Bracis C, Mueller T (2017). Memory, not just perception, plays an important role in terrestrial mammalian migration. Proc R Soc B Biol Sci.

[17] Merkle JA, Sawyer H, Monteith KL, Dwinnell SPH, Fralick GL, Kauffman MJ (2019). Spatial memory shapes migration and its benefits: evidence from a large herbivore. Ecol Lett.

[18] Zubiria Perez A, Bone C, Stenhouse G (2021). Simulating multi-scale movement decision-making and learning in a large carnivore using agent-based modelling. Ecol Modell.

[19] Jones TB, Aplin LM, Devost I, Morand-Ferron J (2017). Individual and ecological determinants of social information transmission in the wild. Anim Behav.

[20] Harten L, Katz A, Goldshtein A, Handel M, Yovel Y (2020). The ontogeny of a mammalian cognitive map in the real world. Science.

[21] Beardsworth CE, Whiteside MA, Capstick LA, Laker PR, Langley EJG, Nathan R, Orchan Y, Toledo S, van Horik JO, Madden JR (2021). Spatial cognitive ability is associated with transitory movement speed but not straightness during the early stages of exploration. R Soc Open Sci.

[22] Carter MID, McClintock BT, Embling CB, Bennett KA, Thompson D, Russell DJF (2020). From pup to predator: generalized hidden Markov models reveal rapid development of movement strategies in a naïve long-lived vertebrate. Oikos.

[23] Hernandez-Montero JR, Reusch C, Simon R, Schoner CR, Kerth G (2020). Free-ranging bats combine three different cognitive processes for roost localization. Oecologia.

[24] Dutour M, Randler C, Bertram S (2021). Mobbing responses of great tits (Parus major) do not depend on the number of heterospecific callers. Ethology.

[25] Coomes JR, McIvor GE, Thornton A (2019). Evidence for individual discrimination and numerical assessment in collective antipredator behaviour in wild jackdaws (*Corvus monedula*). Biol Lett.

[26] Dutour M, Kalb N, Salis A, Randler C (2021). Number of callers may affect the response to conspecific mobbing calls in great tits (Parus major). Behav Ecol Sociobiol.

[27] Carlson NV, Healy SD, Templeton CN (2020). Wild fledgling tits do not mob in response to conspecific or heterospecific mobbing calls. Ibis.

[28] Suzuki TN (2018). Alarm calls evoke a visual search image of a predator in birds. Proc Natl Acad Sci U S A.

[29] Suzuki TN (2020). Other species’ alarm calls evoke a predator-specific search image in birds. Curr Biol.

[30] Lowney AM, Flower TP, Thomson RL, Naguib M (2020). Kalahari skinks eavesdrop on sociable weavers to manage predation by pygmy falcons and expand their realized niche. Behav Ecol.

[31] Palmer MS, Gross A (2018). Eavesdropping in an African large mammal community: antipredator responses vary according to signaller reliability. Anim Behav.

[32] Meise K, Franks DW, Bro-Jorgensen J (2018). Multiple adaptive and non-adaptive processes determine responsiveness to heterospecific alarm calls in African savannah herbivores. Proc R Soc B Biol Sci.

[33] Igic B, Ratnayake CP, Radford AN, Magrath RD (2019). Eavesdropping magpies respond to the number of heterospecifics giving alarm calls but not the number of species calling. Anim Behav.

[34] Gallego-Abenza M, Blum CR, Bugnyar T (2021). Who is crying wolf? Seasonal effect on antipredator response to age-specific alarm calls in common ravens, *Corvus corax*. Learn Behav.

[35] Novakova N, Vesely P, Fuchs R (2020). Object categorization by wild-ranging birds in nest defence. Anim Cogn.

[36] Boogert NJ, Fawcett TW, Lefebvre L (2011). Mate choice for cognitive traits: a review of the evidence in nonhuman vertebrates. Behav Ecol.

[37] Morand-Ferron J (2017). Why learn? The adaptive value of associative learning in wild populations. Curr Opin Behav Sci.

[38] Wetzel DP (2017). Problem-solving skills are linked to parental care and offspring survival in wild house sparrows. Ethology.

[39] Cauchard L, Angers B, Boogert NJ, Lenarth M, Bize P, Doligez B (2017). An experimental test of a causal link between problem-solving performance and reproductive success in wild great tits. Front Ecol Evol.

[40] Shaw RC, MacKinlay RD, Clayton NS, Burns KC (2019). Memory performance influences male reproductive success in a wild bird. Curr Biol.

[41] Rochais C, Pillay N, Schradin C (2021). Do alternative reproductive tactics predict problem-solving performance in African striped mice?. Anim Cogn.

[42] Sonnleitner R, Ringler M, Loretto MC, Ringler E (2020). Experience shapes accuracy in territorial decision-making in a poison frog. Biol Lett.

[43] Bókony V, Pipoly I, Szabó K, Preiszner B, Vincze E, Papp S, Seress G, Hammer T, Likera A (2017). Innovative females are more promiscuous in great tits (Parus major). Behav Ecol.

[44] Ringler E, Szipl G, Harrigan RJ, Bartl-Binder P, Mangione R, Ringler M (2018). Hierarchical decision-making balances current and future reproductive success. Mol Ecol.

[45] Chittka L (2017). Bee cognition. Curr Biol.

[46] Reznikova Z (2020). Spatial cognition in the context of foraging styles and information transfer in ants. Anim Cogn.

