# Supplementary material for: Wild cognition – linking form and function of cognitive abilities within a natural context
Source: Curr Opin Behav Sci. Author manuscript; Available in PMC 2024 Jul 10. (PMC7616152; doi:10.1016/j.cobeha.2022.101115)
Supplement: Supplementary material [file EMS196921-supplement-Supplementary_material.docx]

# Supplementary material

for

Birgit Szabo, Anyelet Valencia-Aguilar, Isabel Damas-Moreira, Eva Ringler (2021) Wild cognition – Linking form and function of cognitive abilities within a natural context. *Current Opinion in Behavioral Sciences*

## Systematic literature search

### Definition of search terms and search in web of science (20^th^ April 2021)

Selected keywords: Cognit*, learn*, memor*, retent*, assess*, discri*, spati*, conditioning, acquis*, experie*, habitu*, “wild”, animal*

Search in topic, all years (1900-2021), all databases

Exact term, syntax by Web of Science:

TOPIC: (Cognit* OR learn* OR memor* OR retent* OR assoss* OR discri* OR spati* OR conditioning OR acquis* OR experie* OR habitu*) AND TOPIC: ("wild") AND TOPIC: (animal*)

References found: 13,588

Further refinement by Web of Science Categories

Only include: Zoology, ecology, neurosciences, behavioural sciences, veterinary sciences, biology, environmental sciences, evolutionary biology, biodiversity conservation, marine freshwater biology, psychology biological, environmental studies, developmental biology, psychology multidisciplinary, entomology, ornithology, psychology experimental, psychology,

References found: 8,097

Further refinement by Publication Years

Only include: 2021, 2020, 2019, 2018 and 2017

References found: 2,629

2,629 references were downloaded and imported into Endnote

### Search in Scopus with the selected keywords (20^th^ April 2021)

Search in article title, abstract, keywords, data range: 2017-present

Exact term, syntax by Scopus

( TITLE-ABS-KEY ( cognit* OR learn* OR memor* OR retent* OR assoss* OR discri* OR spati* OR conditioning OR acquis* OR experie* OR habitu* ) AND TITLE-ABS-KEY ( "wild" ) AND TITLE-ABS-KEY ( animal* ) ) AND PUBYEAR > 2016

References found: 6,842

Further refinement by Subject Area

Only include: Agricultural and biological sciences, Neuroscience, environmental science, multidisciplinary, veterinary, psychology, decision science

References found: 4,523

Further refinement by Keywords

Only include: Animals, animal, nonhuman, mouse, mice, wild population, animal behavior, wild animal, wild, cognition, behaviour, learning, memory, psychology, spatial memory, maze test, bird, maze learning, rat, morris water maze test, aves, comparative study, behavior, primate, spatial learning, birds, mammalia, fish, behavioral response, pig, primates, mammal, decision making, Sus scrofa, survival

References found: 4,083

Further refinement by Keywords

Exclude: Genetics, animal tissue, animal model, human, C57BL mouse, mice, inbred C57BL, animal cell, humans, unclassified drug, protein expression, disease model, disease models animal, pathology, transgenic mouse, knockout mouse, mice transgenic, mice, knockout, drug effect, Alzheimer disease, pathophysiology, nerve cell, gene expression, signal transduction, immunohistochemistry, Alzheimer's disease, amyloid beta protein, nerve cell plasticity, veterinary medicine, chemistry, gene, microbiology, protein function, gene expression regulation, mutation, amyloid precursor protein, conservation of natural resources, upregulation, microglia, virology, cytology, messenger RNA, amyloid beta-peptides, immunology, amyloid beta-protein Precursor, synapse, isolation and purification, real time polymerase chain reaction, protein phosphorylation, gene deletion, in vitro study, inflammation, tau protein, down regulation, environmental monitoring, nervous system inflammation, synaptic transmission, embryo, genetic variation, enzyme linked immunosorbent assay, gene mutation

Reference found: 1,199

1,199 references were downloaded and imported into EndNote

### Search in ProQuest Dissertations & Theses Global with the selected keywords (20^th^ April 2021)

Search in all fields except the full text, last 5 years, all manuscript types and all languages

Exact term, syntax by ProQuest Dissertations & Theses Global

noft(Cognit* OR learn* OR memor* OR retent* OR assess* OR discri* OR spati* OR conditioning OR acquis* OR experie* OR habitu*) AND noft("wild") AND noft(animal*)

References found: 409

Further refinement by topic

Only include: Ecology, biology, neurosciences, animal sciences, zoology, behavioral sciences, wildlife management, wildlife conservation, conservation biology, evolution & development, aquatic sciences, environmental science, evolution and development, veterinary services, developmental biology, agriculture, animal behaviour, behavioral psychology, environmental studies, entomology, morphology, neurobiology, psychology, animals, biological oceanography, evolution, memory, metabolism, psychobiology

Exclude: Genetics, microbiology, physiology, immunology, molecular biology, animal diseases, epidemiology, medicine, pharmacology, aging, biochemistry, cellular biology, biomedical engineering, pathology, endocrinology, public health, virology, bioinformatics, toxicology, archaeology, nutrition, pharmaceutical sciences, proteins, cultural anthropology, remote sensing, Alzheimer’s disease, American literature, computer science, creative writing, gene expression, geography, health sciences, oncology, peptides, physical anthropology, sustainability, women’s studies, acoustics, African studies, American history, biophysics, biostatistics, biosynthesis, cell cycle, climate change, clinical psychology, deoxyribonucleic acid—DNA, education, environmental health, estrogens, genomes, hydrologic sciences, infections, kinases, middle eastern studies, mutation, nanotechnology, native American studies, natural resource management, parasitology, pathogenesis, philosophy, political science, range management, recreation, religion, sociology, statistics, transgenic animals, veterinary medicine, acquired immune deficiency syndrome--aids

References found: 98

98 references were downloaded and imported into EndNote

Overall, 3,926 references were selected (before duplicate removal)

### Duplicate removal using the built in function by EndNote (20^th^ April 2021)

Duplicates removed: 481

Final sample of references after duplicate removal: 3,445

### Selection of references based on Title (23^rd^ April 2021 – 11^th^ May 2021)

Criteria

1. Must mention study was done in animals

2. Must mention study tested cognitive ability (task in which information processing was essential for task success) including innovation and problem-solving

3. Can mention study was done in the wild, wild-caught individuals or individuals kept in semi-natural conditions

References selected: 224

Duplicates removed manually: 82

### Selection of references based on Abstract (11^th^ May 2021 – 17^th^ May 2021)

Criteria

1. Must mention study tested cognitive ability (task in which information processing was essential for task success) including innovation and problem-solving

2. Should mention study was done in the wild, wild-caught individuals or individuals kept in semi-natural conditions

a. If abstract does not specifically mention origin of animals the full text is checked

3. The task tested should indicate an ability that could be of importance in the wild

4. Reviews are separated

References selected: 101

2021: 11

2020: 26

2019: 16

2018: 27

2017: 21

Reviews separated: 38

### Full text download (17^th^ of May 2021)

A full text of all references was obtainable. We focused on the three most recent years 2019, 2020 and 2021.

### Forward and backward searches in the selected references (18^th^ May 2021 – 10^th^ of June 2021)

We performed five forward (references citing the selected paper, search forward in time) and backward (references cited by the selected paper, search backward in time) searches based on the selected references. We used google scholar to perform the forward and backward searches and selected references based on abstract (selection criteria above) and only when they were published in the last five years (2017-2021).

1^st^ forward search (53 references published between 2019-2021) resulted in 16 new references

1^st^ backward (53 references published between 2019-2021) search resulted in 30 new references

2^nd^ forward (46 references from 1^st^ forward and backward search) search resulted in 0 new references

2^nd^ backward (46 references from 1^st^ forward and backward search) search resulted in 4 new references

3^rd^ forward (4 references from 2^nd^ forward and backward search) search resulted in 1 new references

3^rd^ backward (4 references from 2^nd^ forward and backward search) search resulted in 0 new references

4^th^ forward/backward (1 reference from 3^rd^ forward and backward search) search resulted in 1 new references

5^th^ forward/backward (1 reference from 4^th^ forward and backward search) search resulted in 0 new references

A total of 39 new references published between 2019-2021 were found resulting in overall 92 references published in the last three years:

2021: 23

2020: 41

2019: 28

After further check we removed 7 references because they were not original empirical studies but reviews and 1 was a duplicate.

References still included: 84

### Summary of study by cognitive ability tested (10^th^ of June – 11^th^ of June)

After summarising the references by class we discovered a lack of studies in amphibians. We had done a separate systematic search for studies in amphibian cognition and could identify four studies that fit the criteria of the current search. These studies were included to have papers on all vertebrate classes.

Also, we found four studies published in 2021 which were published after our initial search. These studies were also included in the final sample of references.

**Final number of references between 2019 and 2020: 92**

### Full text screening (11^th^ June 2021 – 6^th^ August 2021)

We proceeded to reading the full text of the studies separating them into categories within an excel table (see other supplementary files) which were then used in the review:

- Navigating to find resources
- Predator avoidance
- Reproductive success

The excel table specifies why studies were not included in our final review. We also decided to widen the timeframe of inclusion from the three most recent years to the five most recent years and performed additional searches (forward and backward) to identify further studies that would fit our three major topics published between 2017 and 2021. We added 17 references (2017 = 6 ; 2018 = 6; 2019 = 1; 2020 = 4) of which 11 were included in our review.
